# Research on the Influence of Fibers on the Mechanical Properties of Asphalt Mixtures

**DOI:** 10.3390/ma18214971

**Published:** 2025-10-31

**Authors:** Qinyu Shi, Zhaohui Pei, Keke Lou

**Affiliations:** 1 School of Digital Construction, Shanghai Urban Construction Vocational College, Shanghai 200438, China; klnhsqy@163.com; 2 College of Civil Engineering and Transportation, Yangzhou University, Yangzhou 225127, China; mz220200335@yzu.edu.cn

**Keywords:** asphalt mixture, basalt fiber, crack resistance, fatigue life, dynamic modulus

## Abstract

Fiber reinforcement is a promising solution to several problems, however, the impact of fiber characteristics on the mechanical behavior and reinforcement mechanisms of asphalt mixtures remains unclear. Therefore, two distinct forms of basalt fiber—chopped basalt fiber (CBF) and flocculent basalt fiber (FBF)—were employed. A comprehensive experimental program was conducted, encompassing macroscopic and microscopic analyses through semi-circular bending tests integrated with digital image correlation, four-point bending fatigue tests, and dynamic modulus tests. Results indicate that both fiber types significantly improve crack resistance, with FBF demonstrating superior performance. Compared with the ordinary mixture, the flexibility index and fracture energy of the FBF-reinforced asphalt mixture increased by 59.7% and 30.6%, respectively. Fibers exert a crack-bridging effect, delaying the transition of the crack propagation stage by 1.25–2.21 s and reducing the crack propagation rate by 39.6–55.4%. Although fatigue life decreased with increasing strain levels, basalt fibers substantially enhanced fatigue resistance, with FBF-reinforced asphalt mixture achieving 20–40% higher N_f,50_ values than CBF. Dynamic modulus tests revealed that fibers reduce modulus at low temperatures while increasing it at high temperatures, with more pronounced reinforcement effects observed in high-frequency regions. These findings underscore the importance of fiber morphology in optimizing asphalt mixture design and provide a theoretical basis for optimizing fiber-reinforced pavement materials to achieve long-term durability under complex environmental and traffic load conditions.

## 1. Introduction

Asphalt pavement, as the most prevalent form of pavement in highway transportation systems, plays a critical role in determining driving safety, ride comfort, and the overall economic and social benefits of road infrastructure [1,2]. However, as a typical viscoelastic material, asphalt mixture is inherently susceptible to various distresses under the combined effects of long-term environmental aging and repeated traffic loading [3]. Among these distresses, cracking and fatigue damage are the most common and detrimental [4]. Low-temperature cracking induced by thermal contraction and fatigue cracking resulting from cyclic loading not only compromise the structural integrity of the pavement but also facilitate water infiltration, which accelerates the softening and deterioration of the underlying base layer [5]. This progressive degradation ultimately leads to severe structural failure, significantly reduces service life, and necessitates substantial financial investment for maintenance and rehabilitation [6]. Consequently, the development of high-performance, long-life asphalt pavement materials with enhanced crack and fatigue resistance has become a key focus in road engineering research.

Fiber reinforcement technology is widely recognized as an effective approach to improving the performance of asphalt mixtures [7,8]. The incorporation of fibers can create a three-dimensional network within the matrix, providing multiple reinforcing mechanisms such as bridging, crack resistance, adsorption, and toughening, thereby substantially enhancing the composite mechanical properties of the mixture [9,10]. Early research and practical applications primarily centered on lignin fibers, mineral fibers, and polymer-based fibers. Lignin fibers are complex polymers composed of three main components: lignin, cellulose, and hemicellulose. Valued for their excellent bitumen absorption capacity and thermal stability, they have been extensively used in stone–mastic–asphalt (SMA) mixtures to stabilize binder and prevent drain-off during construction [11]. However, due to their relatively low intrinsic strength, their contribution to mechanical property enhancement remains limited. In contrast, polymer fibers—such as polyester and polypropylene fibers—exhibit superior tensile strength and toughness [12,13], demonstrating more effective improvements in the crack resistance and durability of asphalt mixtures.

In recent years, basalt fiber, as a novel inorganic green fibrous material, has attracted widespread attention due to its superior performance characteristics [14]. As a material derived from molten basalt ore, basalt fiber possesses a combination of properties—including high tensile strength, a high elastic modulus, and superior resistance to temperature, chemicals, and moisture—along with strong asphalt adhesion and low environmental impact [15]. Research confirms that its incorporation into asphalt mixtures leads to significant enhancements in pavement performance [16,17]. The widely accepted reinforcement mechanism is that the three-dimensional network structure formed by the fibers within the mixture effectively redistributes stress, restricts relative aggregate displacement, and absorbs a portion of fracture energy [18]. Long et al. [19] conducted a series of laboratory experiments on asphalt mixtures incorporating varying proportions of basalt fibers and observed that, as the fiber content increased, the dynamic stability of SMA mixtures improved steadily, along with a notable enhancement in crack resistance. Zhang et al. [20] found that basalt fiber outperformed lignin fiber in improving the flexural and tensile properties of asphalt mixtures. For asphalt mixtures, the dosage of basalt fiber is typically low, with a common content range of 0.3% to 0.5% (by total weight of the mixture). Cetin et al. [21] reported that mixtures with 0.4% basalt fiber exhibited the highest resistance to permanent deformation. Kong and Alfalah et al. [22,23] found that basalt fibers provide effective bitumen adhesion and anchorage effects, identifying 0.3% as the optimal content. Through direct tensile tests, Yang et al. [24] investigated the influence of fiber content on mechanical properties such as tensile strength and ultimate strain, establishing 0.5% as the optimum basalt fiber dosage. A significant portion of existing research has concentrated on chopped basalt fibers, typically ranging from 3 to 20 mm in length, whereas limited attention has been devoted to flocculent basalt fibers, another fiber configuration [25]. Flocculent fibers consist of loosely aggregated, highly slender continuous monofilaments, exhibiting a larger specific surface area and more intricate spatial distribution. These characteristics theoretically enable the formation of a denser reinforcement network and facilitate more extensive interaction with asphalt matrix. Wu et al. [26] demonstrated that fibers with irregular morphologies can enhance the interface transition zone between asphalt and aggregates, while Cai et al. [27] indicated that FBF can significantly improve the resilience of asphalt mortar against elastic deformation, thereby improving fatigue resistance. Nevertheless, the quantitative correlation between fiber morphology and key pavement performance indicators remains unclear [28]. Furthermore, the mesoscopic mechanisms by which flocculent fibers influence crack propagation and accommodate thermal variations require further investigation.

It is crucial to select appropriate mechanical property test methods for the scientific and systematic evaluation of fiber reinforcement effects. Although traditional performance tests can provide fundamental performance parameters [29,30], they are limited in their ability to reveal the fracture and fatigue behavior of materials under complex stress conditions. As a simple and effective fracture-mechanics-based method, the evaluation of crack resistance in asphalt mixtures commonly relies on the SCB test, which provides key fracture parameters such as fracture energy and toughness [31]. In recent years, the integration of digital image correlation, a full-field and non-contact optical measurement technique, into SCB testing has emerged as a research focus [32]. DIC technology enables real-time and continuous monitoring of full-field displacement or strain on the specimen surface during testing, allowing precise identification of the crack tip, visualization of crack propagation paths, and quantitative analysis of strain field evolution throughout the cracking process [33]. This provides unprecedented microscopic insights into the fiber “bridging” and “crack resistance” mechanisms. The four-point bending fatigue (FPBF) test is recognized as the most classical method for evaluating fatigue performance both domestically and internationally [34,35]. In this test, the specimen experiences a constant pure bending moment at the bottom of the beam, resulting in a stress state that closely simulates the response of actual pavements under wheel loading. The method yields highly repeatable results and is therefore regarded as a standard approach for investigating the fatigue behavior of asphalt mixtures [36]. The dynamic modulus test serves as a core procedure for characterizing the viscoelastic properties of asphalt mixtures. It measures the dynamic modulus and phase angle of the mixture under varying temperatures and loading frequencies, effectively capturing the mechanical behavior of materials across a wide range, from high-temperature, low-frequency conditions to low-temperature, high-frequency conditions [37]. Given the roles as fundamental input parameters in the mechanistic–empirical pavement design method, these parameters are indispensable for achieving accurate predictions of how fiber-reinforced asphalt pavements will perform structurally under in-service environmental and traffic stresses [38].

Therefore, in this study, the fiber characteristic was adopted as a key variable and to systematically evaluate the influence of chopped basalt fiber (CBF) and flocculent basalt fiber (FBF) on the mechanical properties of asphalt mixtures by integrating the SCB test, DIC technology, FPBF test, and dynamic modulus test. The underlying mechanisms of action were thoroughly investigated, addressing gaps in existing research regarding fiber types and morphological characteristics. This work aims to provide a theoretical foundation for the precise application and performance enhancement of basalt fibers in asphalt pavements, thereby promoting the advancement of long-life pavement technologies.

## 2. Materials and Test Methods

### 2.1. Raw Materials and Sample Preparation

#### 2.1.1. Fiber

**Figure 1:**
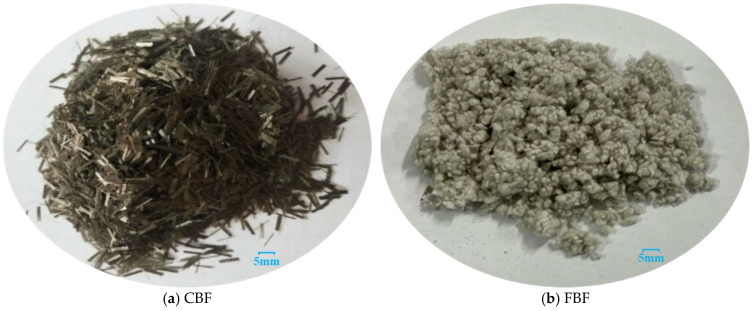
Macroscopic morphology images of basalt fiber.

Two different types of basalt fibers, CBF and FBF, were selected in this study, as shown in Figure 1. CBF features a simple, linear topology and a regular, cylindrical morphology. In contrast, FBF possesses a complex, three-dimensional network topology and an irregular, fluffy morphology. Specifically, the CBF exhibits a uniform chopped structure, with a fixed length. In contrast, the FBF presents as cotton-like agglomerates with irregular particle sizes. The technical parameters of basalt fiber are listed in Table 1. These data are provided by the manufacturer Jiangsu Tianlong Basalt Continuous Fiber Co., Ltd. (Yizheng, China), which meet the requirements of specification JTT 533 [39].

#### 2.1.2. Asphalt

In this study, the asphalt binder employed in this study was SBS-modified and graded as PG76-22, the softening point of the asphalt was 86 °C, the penetration was 71 (0.1 mm), and the ductility (5 cm/min, 5 °C) was 48 cm.

#### 2.1.3. Gradation Design

**Figure 2:**
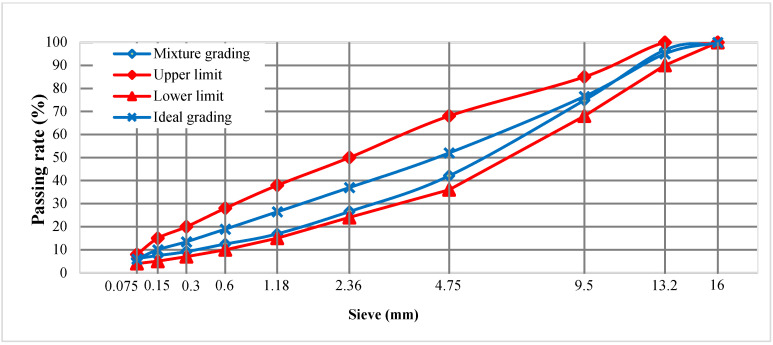
Gradation curves.

The dense-graded gradation of AC-13 was designed and prepared using the Marshall method, and the gradation curves are shown in Figure 2. Based on the previous research results of the project team, the dosages of both CBF and FBF were uniformly fixed at 0.3% by the weight of the mixture [25,40]. The specimens were molded through the Marshall test in accordance with the standard JTG E20 [41]. These involved testing specimens at different asphalt contents (OACs) and selecting the optimum based on the analysis of stability, flow, air voids, and voids filled with asphalt. The corresponding OACs for the asphalt mixture without fibers (AC-13), the asphalt mixture with CBF (CBFAC-13), and the asphalt mixture with FBF (FBFAC-13) were 4.9%, 5.1%, and 5.2% by weight of the mixture, respectively.

### 2.2. Test Methods

#### 2.2.1. SCB Test

According to AASHTO T393 [42], samples with a height of 115 mm and a diameter of 150 mm were prepared using a laboratory rotary compaction apparatus, and then the SCB semi-circular flexural specimens with a thickness of 50 mm were obtained by cutting. The crack resistance of asphalt mixtures was evaluated via the SCB test at 25 °C. The specimens were fabricated into a semi-circular geometry with dimensions of 150 mm in diameter and 50 mm in thickness. A 15-mm-long notch was introduced at the bottom center of each specimen to simulate an initial crack. Testing was performed on a UTM-25 machine (IPC Global, Boronia, Victoria, Australia) with a constant vertical loading rate of 50 mm/min. The evaluation relied on two quantitative indicators: fracture energy (*G_f_*) and flexibility index (*FI*), derived from Equations (1) and (2), respectively.

(1) $$G_{f}=\frac{W_{f}}{Area_{lig}}\times 10^{6}$$

(2) $$FI=\frac{G_{f}}{\left|m\right|}\times A$$

where *W_f_* denotes the work of fracture, J; *Area_lig_* is ligament area, mm^2^; $\left|m\right|$ represents the absolute value of post-peak load slope, kN/mm; *A* is a unit conversion and scaling factor set to 0.01.

#### 2.2.2. DIC Technology

**Figure 3:**
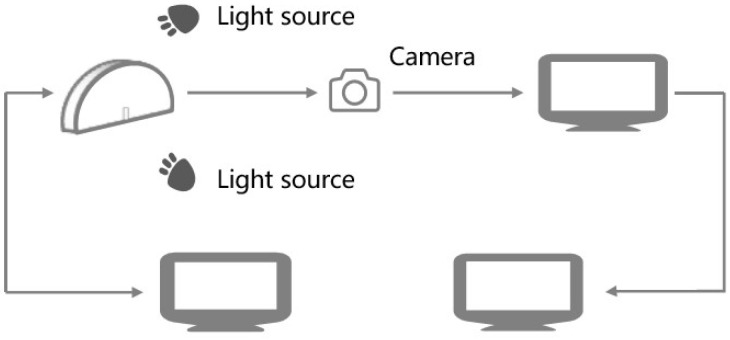
Schematic diagram of image data information collection.

**Figure 4:**
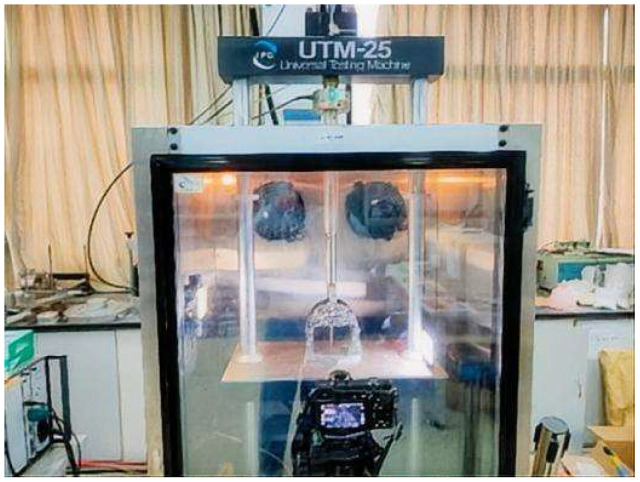
Experimental process diagram of SCB test with DIC.

DIC technology measures surface deformation of objects using optical methods. The fundamental principle involves the automatic identification and tracking of pixel points on the specimen surface during the deformation process. By measuring and analyzing variations in gray-level patterns within adjacent local regions, displacement and strain distributions can be determined. In this study, DIC was employed exclusively during the monotonic SCB test to quantitatively analyze the surface deformation and crack propagation behavior. A single high-resolution CCD camera of SONY α 6400 (Tokyo, Japan) was utilized to capture the in-plane displacement fields on the specimen surface, with two supplementary lights to ensure adequate lighting conditions, as shown in Figure 3. And the camera was aligned perpendicular to the specimen surface to measure in-plane deformations (displacements Ux and Uy), with the Y-axis defined as the direction of loading. It is important to note that the natural texture and contrast of the fractured aggregate surface were deemed sufficient for the correlation algorithm, thus no artificial speckle pattern was applied. Prior to testing, the surface of the specimen must be polished to produce a distinct and naturally textured surface, which facilitates subsequent digital image processing, and the process of data collection is illustrated in Figure 4. The analysis of deformation fields was performed using the VIC-2D software (Version 6.0). The computation of sub-pixel displacement, along with grayscale value and its gradient, was achieved through eighth-order spline interpolation and the Newton–Raphson iterative method. The deformation fields were studied using the VIC-2D software, with a fixed sub-set size of 71 pixels and a step size of 7 pixels.

#### 2.2.3. Fatigue Test

**Figure 5:**
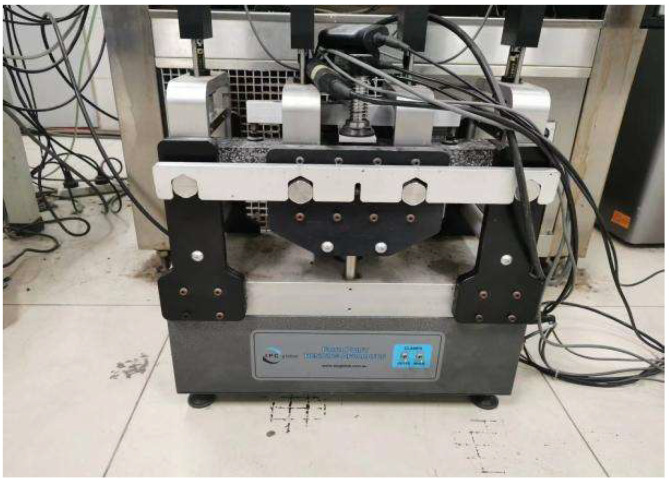
Four-point bending fatigue test.

To evaluate the fatigue performance, FPBF tests were conducted on the asphalt pavement specimens utilizing a strain-controlled mode. According to the standard JTG E20 [41], specimens with dimensions of 400 mm × 300 mm × 75 mm were first fabricated using a wheel compaction method. Subsequently, these specimens were cut into prismatic beams measuring 380 mm × 63.5 mm × 50 mm, with the compaction direction aligned parallel to the length of the beams for testing. Cyclic alternating loads were applied continuously to small beam specimens at strain levels of 450, 650, and 850 με, respectively, until the flexural stiffness modulus of the asphalt mixture dropped to 50% of its initial value, and the samples are shown in Figure 4. The tests were conducted using a UTM-25 testing machine, with the loading configuration illustrated in Figure 5. Fatigue life (*N_f_*) was employed as the key indicator to assess the fatigue resistance of the asphalt mixtures, and the corresponding calculation formula is provided in Equation (3).

(3) $$N_{f}=c{\left(\frac{1}{\varepsilon }\right)}^{m}$$

where *ε* is the maximum amplitude of the constant strain applied to the specimen each time; *c* and *m* are parameters associated with the composition and properties of asphalt mixtures.

#### 2.2.4. Dynamic Modulus Test

**Figure 6:**
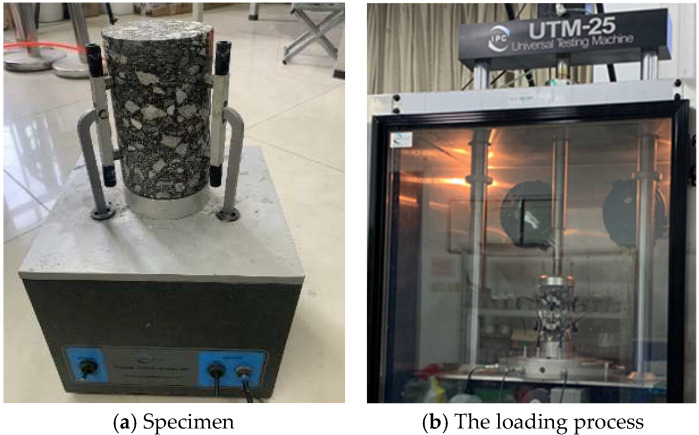
The dynamic modulus test.

The specimens were fabricated using a gyratory compactor in accordance with standard JTG E20 [41], with initial dimensions of 150 mm in diameter and 170 mm in height. Core samples of 100 mm in diameter were then drilled from these specimens and precisely trimmed at both ends to achieve the final test height of 150 ± 2.5 mm. To fully characterize the viscoelastic properties, the dynamic modulus of the fiber-reinforced asphalt mixtures was evaluated using a repeated loading test protocol. The experimental matrix encompassed a broad range of service conditions, including five temperatures (50, 35, 20, 5, and −10 °C) and six loading frequencies (25, 10, 5, 1, 0.5, and 0.1 Hz). In the test, unconfined cylindrical specimens (as illustrated in Figure 6a) were subjected to a continuous sinusoidal axial compressive stress. The resultant strain response was measured, enabling the calculation of two key viscoelastic parameters: the dynamic modulus, which indicates the material’s ability to resist deformation, and the phase angle, which reflects its viscous energy dissipation characteristics. A schematic of the loading configuration is provided in Figure 6b. Using |E*| as the key indicator for evaluating the dynamic modulus of asphalt mixtures, the calculation formulas are as shown in Equations (4)–(6).

(4) $$\sigma_{0}=\frac{P_{i}}{A}$$

(5) $$\varepsilon_{0}=\frac{\Delta_{i}}{l_{0}}$$

(6) $$\left|E*\right|=\frac{\sigma_{0}}{\varepsilon_{0}}$$

where *σ*_0_ is the amplitude of the axial stress; *P_i_* is the average amplitude of the axial test load in the last 5 loading cycles; *A* is the radial cross-sectional area of the specimen; *ε*_0_ is the amplitude of axial strain; Δ*_i_* is the average amplitude of recoverable axial deformation over the last 5 loading cycles; *l*_0_ is the measurement interval of the displacement sensor on the specimen; |E*| represents the dynamic modulus of asphalt mixture.

## 3. Experimental Results and Analysis

### 3.1. Results of SCB Test

**Figure 7:**
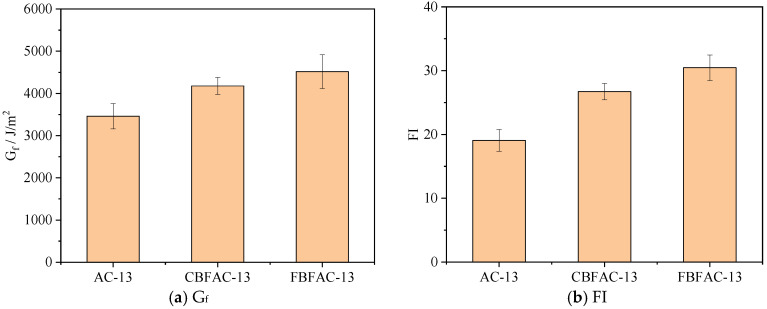
The results of SCB test.

The results of the semi-circular bending test are illustrated in Figure 7. As shown in Figure 7a, compared with the ordinary asphalt mixture, the fracture energy (*G_f_*) of the mixture increased by 20.7% and 30.6%, respectively, after the incorporation of CBF and FBF. This finding confirms that basalt fibers exhibit a favorable reinforcing effect on the crack resistance of dense-graded asphalt mixtures, enabling the mixture to resist cracking more effectively under the same load conditions. As shown in Figure 7b, the addition of basalt fibers led to varying degrees of increase in the flexibility index (*FI*) of the asphalt mixture. Specifically, the *FI* values of the CBFAC-13 and FBFAC-13 asphalt mixtures increased by 40.1% and 59.7%, respectively. Notably, FBF demonstrated a more pronounced toughening and crack-resistant effect on the asphalt mixture than CBF. The explanation could be that FBF exhibits a higher oil absorption capacity than CBF, allowing it to stabilize a greater amount of asphalt. A higher asphalt–aggregate ratio further contributes to enhancing the toughness of the asphalt mixture. Moreover, FBF can take advantage of the high strength of the fiber, and it not only stabilizes substantial amounts of free asphalt but also imparts a certain reinforcing effect to the mixture. Consequently, the final crack resistance performance of the FBF-reinforced asphalt mixture was found to be ultimately more resistant to cracking than that of the mixture reinforced by CBF.

### 3.2. Results of DIC

**Figure 8:**
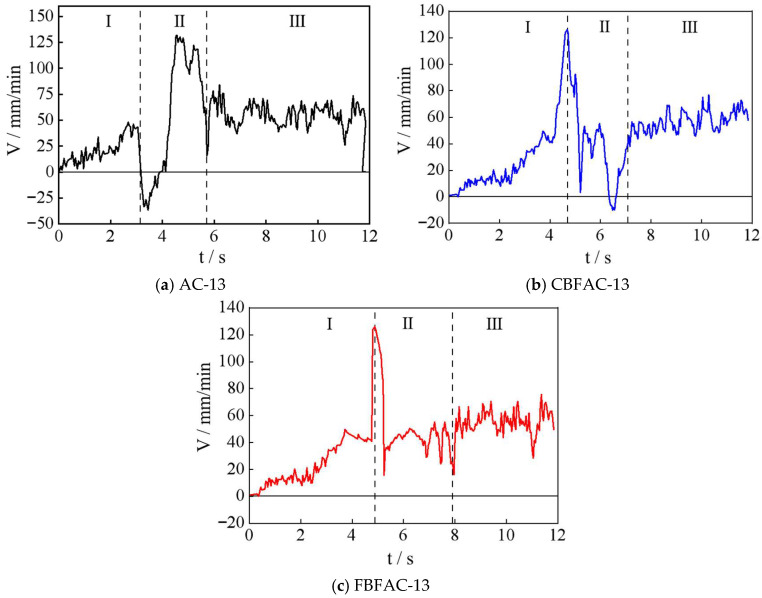
The relationship between the lateral displacement velocity and the loading time.

**Figure 9:**
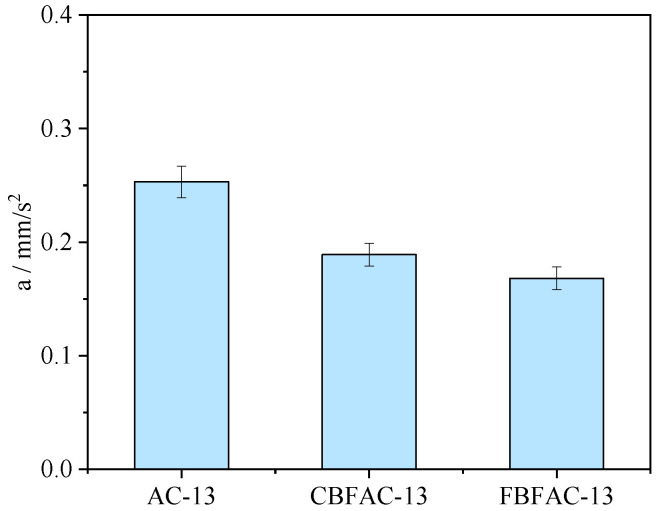
The rate of change of cracking velocity in stage I.

The DIC and SCB test were conducted to record the dynamic cracking process of different types of asphalt mixtures. Figure 8 illustrates the relationship between the lateral displacement velocity and the loading time, and it can be observed that the crack propagation rate of the asphalt mixture exhibits three distinct stages regardless of the presence of fiber. In stage I, the crack propagation rate increases steadily. In stage II, the rate experiences significant fluctuations. In stage III, the cracking rate stabilizes. Specifically, in this study, during the cracking stage III, the rate curve fluctuates within a relatively stable range of 50–60 mm/min, the results in the average cracking rate of stage III being consistent with the loading rate. This phenomenon indicates that the specimen has suffered complete failure. As shown in Figure 8, after the addition of CBF and FBF, the transition time points of cracking stage I and stage II of the asphalt mixture were delayed by 1.25 s and 1.75 s, the transition time points of cracking stage II and stage III of the asphalt mixture were delayed by 1.43 s and 2.21 s, as shown in Figure 9, and the crack propagation rate was reduced by 39.6% and 55.4%, respectively. This indicates that the addition of fibers can significantly slow down the crack development rate, and FBF exhibits a superior effect. The explanation could be that the adhesion between fibers and asphalt in the mixture enables the system to withstand a certain degree of stress. When microcracks initiate, the fibers exert a bridging effect. During the process of fiber–asphalt interface sliding or fiber fracture, external energy is dissipated, and this energy consumption helps delay the propagation of cracks.

### 3.3. Results of Fatigue Test

**Figure 10:**
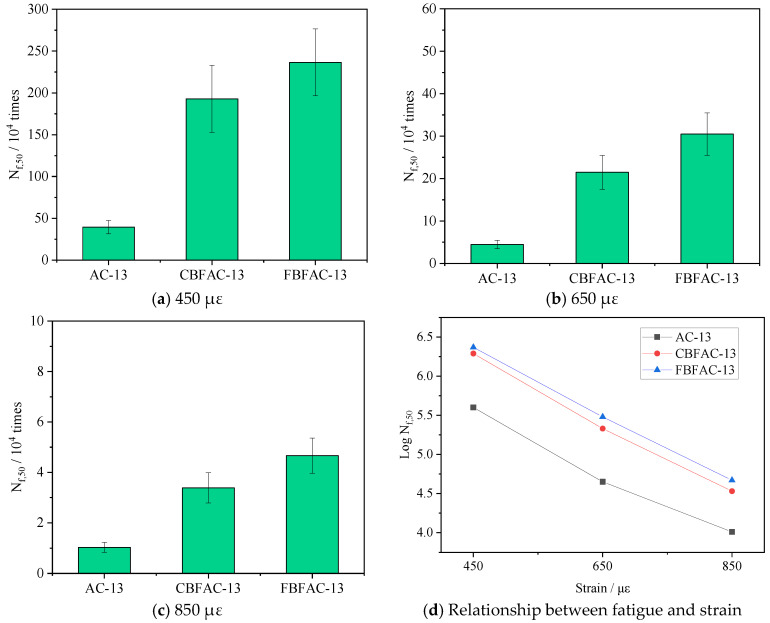
Four-point bending beam fatigue test results.

The results of the four-point bending beam fatigue test for different types of asphalt mixtures are presented in Figure 10. It is evident that the fatigue life of the mixtures decreases with an increase in controlled strain. Specifically, higher strain levels lead to a reduction in the number of fatigue cycles for asphalt mixtures, thereby shortening their overall fatigue life. A significant boost in fatigue life is observed in Figure 10a for basalt-fiber-reinforced mixtures relative to the conventional mixture. This enhancement is particularly pronounced at 450 µε, where the CBF and FBF mixtures exhibited a remarkable 388% and 498% increase in *N_f,_*_50_, underscoring the remarkable efficacy of fiber reinforcement. As shown in Figure 10b, *N_f,_*_50_ of CBF- and FBF-reinforced asphalt mixture increased by 381% and 581% at the strain level of 650 με. As shown in Figure 10c, *N_f,_*_50_ of CBF- and FBF-reinforced asphalt mixture increased by 227% and 351% at the strain level of 850 με. A comparison between CBF-reinforced asphalt mixtures and FBF-reinforced asphalt mixtures reveals that FBF exhibits a superior reinforcing effect. At strain levels of 450 με, 650 με, and 850 με, the *N_f,_*_50_ of FBF-reinforced asphalt mixtures is 22.6%, 41.9%, and 37.8% higher than that of CBF-reinforced asphalt mixtures, respectively. This indicates that FBF provides a more notable reinforcing effect in asphalt mixtures with a suspended dense structure. Both types of basalt fibers possess relatively high tensile strength, which contributes to their reinforcing capacity. Among them, FBFs have a much larger specific surface area, forming an extensive wetting interface with the asphalt binder. The expanded fiber–asphalt interface promotes increased binder adhesion, which reduces the proportion of free asphalt in the mixture and thereby improves its overall structural stability and fatigue resistance. Subsequent analysis of the fatigue life curves plotted for different strain levels (in Figure 10d) reveals that the incorporation of fibers significantly alters the regression parameters (in Table 2). Specifically, an approximately 12% increase in the intercept of the fatigue lines suggests that fibers effectively decelerate the fatigue decay process. Furthermore, the steeper slopes of these lines indicate a more pronounced improvement in fatigue life under lower strain conditions.

### 3.4. Results of Dynamic Modulus Test

**Figure 11:**
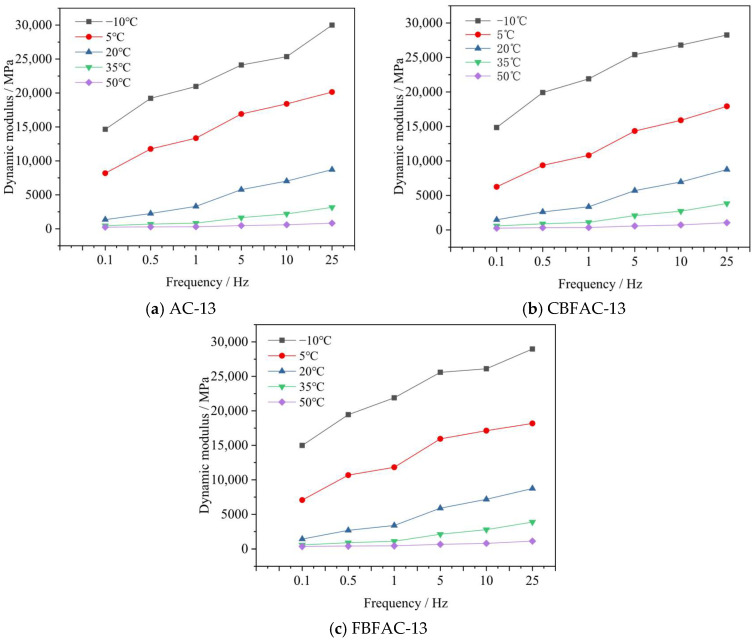
Dynamic modulus.

**Figure 12:**
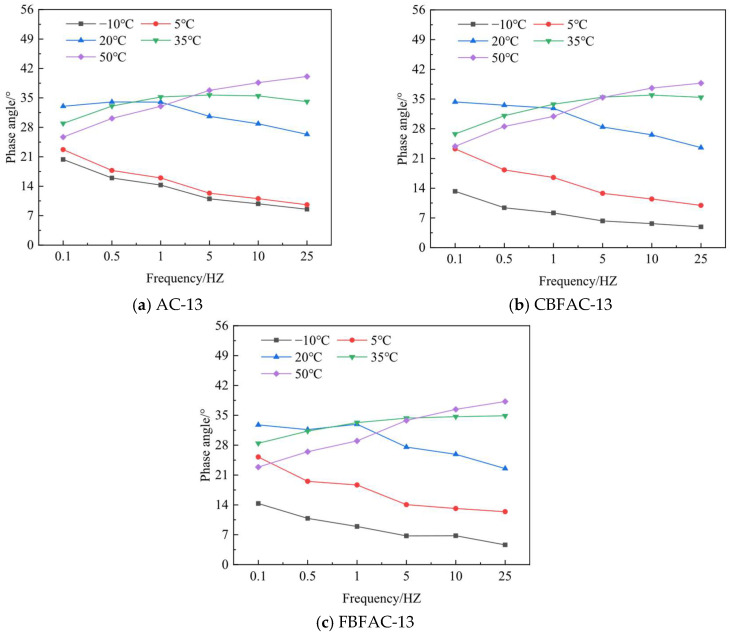
Phase angle.

The viscoelastic characteristics of the asphalt mixtures, represented by the dynamic modulus and phase angle, are detailed in Figure 11 and Figure 12. A fundamental viscoelastic behavior is observed in Figure 11: the dynamic modulus increases with loading frequency due to the reduced relaxation time available for the asphalt binder, while it decreases with rising temperature as the binder softens, reducing the mixture’s overall resistance to deformation. Although both CBF and FBF modifications alter the absolute values of the dynamic modulus, the composites consistently follow this inherent trend. A notable finding is the reduction in modulus caused by fiber addition at low temperatures. This phenomenon may be attributed to the intricate interplay between the stiff fibers and the relatively brittle asphalt matrix at these conditions, potentially introducing new viscoelastic relaxation mechanisms that slightly compromise the composite material’s ability to resist deformation. Specifically, at −10 °C and 25 Hz, the dynamic modulus decreased by 1736 MPa and 1020 MPa, respectively. This can be interpreted as the fibers taking on part of the internal stress of the asphalt mixture after being added, reducing the stress concentration at the crack tip at low temperatures. This improvement in the stress state enhances the flexibility of the asphalt mixture and ultimately boosts its low-temperature deformation capacity. On the contrary, the addition of fibers increased the dynamic modulus of the asphalt mixtures under high-temperature conditions. Specifically, at 50 °C and 25 Hz, the dynamic modulus increased by 218.6 MPa and 285.6 MPa, respectively. A similar increasing trend in dynamic modulus is also observed for fiber-reinforced asphalt mixtures under other loading frequencies. Furthermore, FBF exhibits a superior effect in enhancing both the high-temperature and low-temperature performance of asphalt mixtures compared to CBF. This advantage is attributed to FBF’s higher tensile strength and oil absorption rate, as the higher oil absorption rate helps stabilize the asphalt–aggregate interface, while the higher strength reinforces the mixture’s internal structure, collectively enabling the formation of a more stable asphalt mixture system.

Figure 12 presents the phase angle of the basalt-fiber-reinforced asphalt mixtures under various temperatures and loading frequencies. A key observation is the consistent reduction in phase angle upon fiber addition. This decrease signifies a fundamental shift in the material’s viscoelastic behavior in which the composite exhibits a more pronounced elastic response relative to its viscous behavior. Essentially, the fiber reinforcement restricts the viscous flow of the asphalt binder, thereby enhancing the elastic recovery capability of the mixture. On the one hand, fibers themselves are elastic bodies and, on the other hand, fibers can adsorb the light components of asphalt, thereby altering its viscoelasticity.

**Figure 13:**
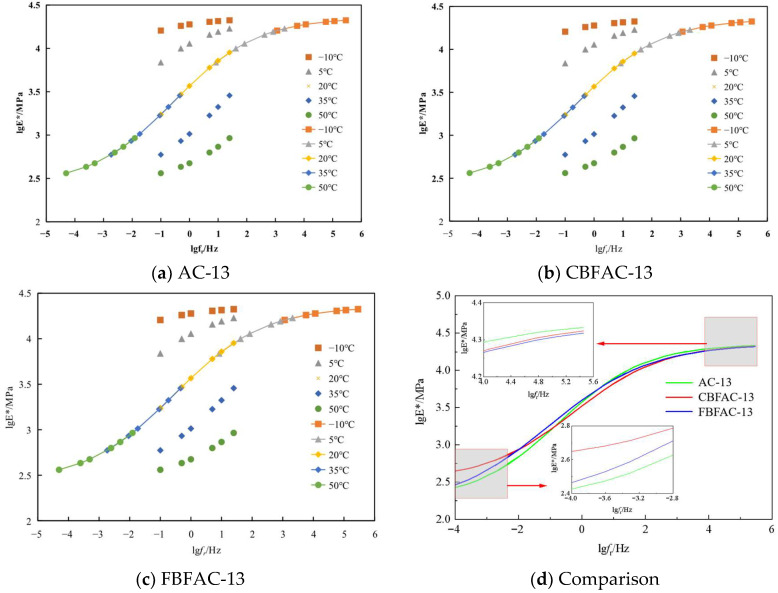
Dynamic modulus master curves.

The dynamic modulus master curves for the various asphalt mixtures are constructed in Figure 13 based on the time–temperature superposition principle. This principle establishes the equivalence between the mechanical responses under high-frequency/low-temperature and low-frequency/high-temperature conditions. The master curves vividly depict the transition in the material’s rheological state: at the high-frequency/low-temperature end, the behavior is predominantly elastic, characterized by a high dynamic modulus and minimal deformation, as molecular chain movement is restricted. Conversely, at the low-frequency/high-temperature end, viscous flow becomes dominant, resulting in a significantly lower modulus and larger deformations, governed by unhindered molecular relaxation. Therefore, the low-frequency and high-frequency regions of the dynamic modulus master curve for asphalt mixtures can be used to evaluate the high-temperature performance and low-temperature performance of asphalt mixtures, respectively. Analysis of the master curves in Figure 13 reveals that basalt fibers contribute to a more balanced pavement performance. The elevated dynamic modulus in the high-frequency domain translates to greater resistance to permanent deformation under high-temperature conditions. Simultaneously, the reduced modulus in the low-frequency domain reflects an increased flexibility, which mitigates low-temperature cracking. This comprehensive enhancement underscores the value of fiber reinforcement in optimizing asphalt mixtures for diverse climatic conditions.

A comparative analysis of the master curves reveals a nuanced reinforcing effect: the improvement in the high-frequency region is more pronounced than that in the low-frequency region. Specifically, the FBF-modified mixture exhibits a higher dynamic modulus than its CBF-modified counterpart at high frequencies, a trend that is conversely observed at low frequencies. This performance inversion confirms that FBF possesses a superior capability to concurrently regulate both the high- and low-temperature viscoelastic properties of the asphalt mixture. The underlying mechanism is attributed to the greater specific surface area of FBF, which facilitates the adsorption of a larger amount of free asphalt, thereby increasing the proportion of structural asphalt. At low temperatures, this enhanced fiber–matrix interaction allows the FBF to more effectively bear internal stress, thereby increasing the flexibility of the asphalt matrix and ultimately optimizing its low-temperature crack resistance.

## 4. Conclusions

The objective of this paper was to investigate the influence of fibers on the mechanical properties of asphalt mixtures, including crack resistance, fatigue resistance, and dynamic modulus. It also investigates the impact of fiber characteristics on these properties. The main conclusions can be drawn as follows.

(1) Both CBF and FBF can significantly improve the crack resistance of asphalt mixtures, among which FBF exhibits the most pronounced reinforcing effect. Specifically, compared with AC-13 asphalt mixture, the *G_f_* and *FI* of FBF-reinforced asphalt mixture increased by 30.6% and 59.7%, respectively, while CBF-reinforced asphalt mixture only improves these two metrics by 20.7% and 40.1%.
(2) Fibers provide effective crack-bridging effects, delaying crack propagation and extending the total time to failure. Notably, flocculent basalt fibers demonstrate a more pronounced capability in reducing the acceleration of crack development during the stable propagation stage.
(3) Fatigue life exhibits a strong inverse correlation with the applied strain amplitude in asphalt mixtures. Basalt fibers markedly improved fatigue resistance, particularly under higher strain amplitudes. FBFAC-13 exhibited 20–40% longer fatigue life than CBFAC-13, highlighting FBF’s advantage in sustaining cyclic loading.
(4) Both fibers positively influenced viscoelastic properties by reducing dynamic modulus at low temperatures while enhancing it at high temperatures. The reinforcement was more pronounced in the high-frequency (low-temperature) domain, demonstrating the fibers’ ability to comprehensively optimize performance across a broad range of pavement service conditions.

## Figures and Tables

**Table 1:** Physical and mechanical properties of basalt fiber.

Properties	CBF	FBF
Diameter/μm	16 ± 0.7	10~20
Length/mm	6 ± 0.5	4~7
Density/g·cm^−3^	2.76	2.82
Oil absorption rate/%	104	230
pH	7.1	7.4

**Table 2:** Fatigue equation of different asphalt mixtures.

Types of Asphalt Mixture	Fatigue Equation	R^2^
AC-13	logN_f_ = 7.3371 − 0.0039 με	0.987
CBFAC-13	logN_f_ = 8.2433 − 0.0044 με	0.997
FBFAC-13	logN_f_ = 8.2692 − 0.0042 με	0.999

## Data Availability

The original contributions presented in this study are included in the article. Further inquiries can be directed to the corresponding author.

## Nomenclature

The following notations were used in this paper:

CBF	Chopped basalt fiber
FBF	Flocculent basalt fiber
SCB	Semi-circular bending
DIC	Digital image correlation
SMA	Stone–mastic–asphalt
FPBF	Four-point bending fatigue
AC-13	Dense-graded asphalt concrete with 13.2 mm nominal maximum aggregate size
CBFAC-13	AC-13 with chopped basalt fiber
FBFAC-13	AC-13 with flocculent basalt fiber
